# Improved Oral Bioavailability Using a Solid Self-Microemulsifying Drug Delivery System Containing a Multicomponent Mixture Extracted from *Salvia miltiorrhiza*

**DOI:** 10.3390/molecules21040456

**Published:** 2016-04-08

**Authors:** Xiaolin Bi, Xuan Liu, Liuqing Di, Qiang Zu

**Affiliations:** College of Pharmacy, Nanjing University of Chinese Medicine, 138 Xianlin Avenue, Nanjing 210023, China; bxl77@126.com (X.B.); ptziye@163.com (X.L.); zuqiang1975@163.com (Q.Z.)

**Keywords:** solid self-microemulsifying drug delivery system, salvia extract, dissolution rate, bioavailability, oral drug delivery

## Abstract

The active ingredients of salvia (dried root of *Salvia miltiorrhiza*) include both lipophilic (e.g., tanshinone IIA, tanshinone I, cryptotanshinone and dihydrotanshinone I) and hydrophilic (e.g., danshensu and salvianolic acid B) constituents. The low oral bioavailability of these constituents may limit their efficacy. A solid self-microemulsifying drug delivery system (S-SMEDDS) was developed to load the various active constituents of salvia into a single drug delivery system and improve their oral bioavailability. A prototype SMEDDS was designed using solubility studies and phase diagram construction, and characterized by self-emulsification performance, stability, morphology, droplet size, polydispersity index and zeta potential. Furthermore, the S-SMEDDS was prepared by dispersing liquid SMEDDS containing liposoluble extract into a solution containing aqueous extract and hydrophilic polymer, and then freeze-drying. *In vitro* release of tanshinone IIA, salvianolic acid B, cryptotanshinone and danshensu from the S-SMEDDS was examined, showing approximately 60%–80% of each active component was released from the S-SMEDDS *in vitro* within 20 min. *In vivo* bioavailability of these four constituents indicated that the S-SMEDDS showed superior *in vivo* oral absorption to a drug suspension after oral administration in rats. It can be concluded that the novel S-SMEDDS developed in this study increased the dissolution rate and improved the oral bioavailability of both lipophilic and hydrophilic constituents of salvia. Thus, the S-SMEDDS can be regarded as a promising new method by which to deliver salvia extract, and potentially other multicomponent drugs, by the oral route.

## 1. Introduction

**Figure 1:**
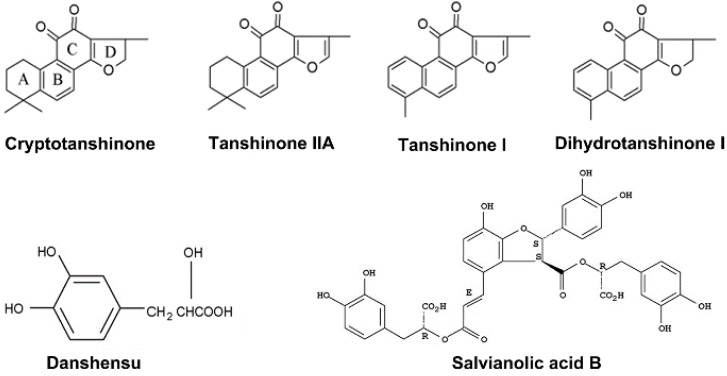
Chemical structures of cryptotanshinone, tanshinone IIA, tanshinone I, dihydrotanshinone I, danshensu and salvianolic acid B.

Salvia is the dried root or rhizome of *Salvia miltiorrhiza* Bunge, which is part of the Labiatae family of flowering plants. The active ingredients of salvia can be divided into two major groups (Figure 1): lipophilic diterpenoid quinone constituents, including tanshinone IIA, tanshinone I, cryptotanshinone and dihydrotanshinone I; and hydrophilic phenolic acid compounds, including danshensu, salvianolic acid B and protocatechuic aldehyde [1]. Salvia has been reported to have a wide range of pharmacological effects, such as improving the coronary circulation, protecting against myocardial ischemia and myocardial infarction, and exerting anti-bacterial, anti-inflammatory, anti-oxidative and anti-tumor actions, in addition to other multi-organ protective effects [1,2,3,4,5]. The numerous components of *S. miltiorrhiza* are thought to have synergistic effects [6,7]. However, diterpenoid quinone compounds have poor water solubility, while hydrophilic phenolic acids have poor stability and permeability, and are regulated by P-glycoprotein [8]. The relevant studies accounting for this are as follows. Low aqueous solubility and poor membrane permeability may be responsible for the poor absorption of tanshinone IIA [9]. A mechanism underlying the poor intestinal absorption of cryptotanshinone in rats is the presence of an efflux pump (P-glycoprotein) on intestinal epithelial cells that transports the drug to the luminal side [10]. Danshensu and salvianolic acid B are extremely hydrophilic compounds with low permeabilities, and as a result have poor oral bioavailabilities (11.09% and 3.90%, respectively); their absorption is predominantly via the paracellular route [11]. The low oral bioavailability of some of its constituents has a detrimental effect on the clinical application of salvia, since some of its multiple active components will show limited absorption.

A variety of approaches have been used to improve the solubility and dissolution properties of fat-soluble compounds, such as the inclusion of β-cyclodextrin [12] and the use of solid dispersions [9,13]. In addition, the permeability of salvianolic acid B has been enhanced through the preparation of a phospholipid complex [14,15]. However, in view of the fact that the Chinese medicinal herbs exert their pharmacological effects in a multi-component and multi-target way, there has been limited success in the development of techniques that can simultaneously improve the bioavailability of both hydrophilic and hydrophobic drugs of salvia in a multicomponent drug delivery system.

Over the last two decades, self-microemulsifying drug delivery systems (SMEDDS) have been investigated in numerous studies as a potentially effective means of delivering poorly water-soluble drugs via the oral route. These systems are composed of oil, surfactant and cosurfactant. Upon mild agitation followed by dilution in an aqueous medium (which can include gastrointestinal fluids), this system can form fine oil-in-water (o/w) microemulsion droplets [16]. The resultant small droplet size provides a large surface area for drug release and absorption [17]. In addition, the absorption of both hydrophobic and hydrophilic drugs can be enhanced by increasing the membrane fluidity to facilitate transcellular absorption, opening tight junctions to allow paracellular transport, and inhibiting efflux pumps like P-glycoprotein [18]. At the very beginning, SMEDDS was introduced in order to improve the oral bioavailability of both hydrophilic and hydrophobic drugs of salvia in our research. However, hydrophilic drugs could be loaded very little. Considering the formulations of SMEDDS, it has other drawbacks, such as a smaller range of dosage forms, low manageability and portability, and a limited shelf-life (owing to problems of interactions between the constituents and/or oxidation of lipid components during storage), as well as possible compatibility issues and risks of leakage when the formulations are filled into hard gelatin capsules [19,20]. To address such critical challenge, here we report a solid self-microemulsifying drug delivery system that was developed to allow the various constituents of salvia to be loaded into a single drug delivery system and improve the oral bioavailability of both hydrophilic and hydrophobic compounds.

Solid self-microemulsifying drug delivery system (S-SMEDDS) is one of the lipid-based drug delivery systems prepared by the incorporation of liquid excipients into powders by solidification. It combines the advantages of liquid SMEDDS (solubility and bioavailability enhancement) with those of solid dosage forms (high stability with various dosage forms options) [21,22]. Solid SMEDDS produce oil-in-water microemulsions with droplet sizes of less than 200 nm upon mild agitation in aqueous media (such as gastrointestinal fluids) [22,23]. These fine microemulsion droplets have the advantage of presenting the drug in a dissolved form with a large interfacial surface area for drug absorption, which results in an enhanced and more uniform and reproducible bioavailability [24].

In order to achieve this aim, prototype formulations of a SMEDDS were generated, and S-SMEDDS was prepared by freeze drying, using a mixture of mannitol and Dextran-40. The drug release characteristics and bioavailability of the novel S-SMEDDS were then assessed.

## 2. Results and Discussion

### 2.1. Preparation of the Liquid and Solid SMEDDS

In order to obtain an optimized formulation of the SMEDDS, we determined the solubility of the salvia extract in various oils, surfactants and cosurfactants. The results are presented in Table 1. The solubilities of the liposoluble and aqueous extracts in the oils and surfactants tested varied greatly. Overall, among the oils the highest solubilities were observed for Maisine 35-1 and isopropyl myristate (IPM), as well as Cremophor RH40 and Solutol HS15 (among the surfactants). The solubility of salvia extract in Transcutol P was markedly higher than that in the other cosurfactants. Based on these results, we selected for further investigation Maisine 35-1 and IPM as the oil phase, Cremophor RH40 and Solutol HS15 as the surfactants, and Transcutol P as the cosurfactant.

**Figure 2:**
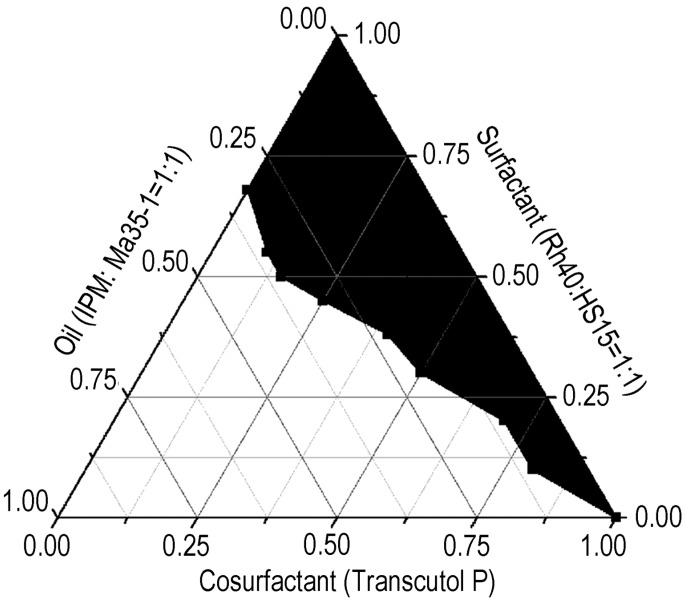
Ternary phase diagram for the mixture containing Oil:IPM/Maisine 35-1 (1:1), surfactant: Cremophor-RH40/Solutol HS15 (1:1) and cosurfactant: Transcutol P. The black domain indicates the SMEDDS region.

A phase diagram was constructed to identify the self-microemulsification regions. A series of formulations containing Maisine 35-1, IPM, Cremophor RH40, Solutol HS15 and Transcutol P were prepared and diluted 100-fold in distilled water at 37 °C. A dispersion with a transparent appearance was regarded as a microemulsion. The phase diagram is shown in Figure 2.

Based on the analysis of the self-microemulsifying regions, five formulations were selected for further assessment of droplet size, polydispersibility and equilibrium solubility. As shown in Table 2, the droplet size of formulation E (containing Maisine 35-1+ IPM (1:1), Cremophor RH40+ Solutol HS15 (1:1) and Transcutol P) was clearly smaller than those of the other formulations (which were composed of only three of these vehicles). These data are consistent with a previous study in which we found that the droplet size of the formulation could be reduced by using a mixture of two types of oil or two types of surfactant [25]. In addition, formulation E showed a favorable solubilizing capacity (Table 2). Based on these findings, formulation E was selected as the final prototype SMEDDS.

**Figure 3:**
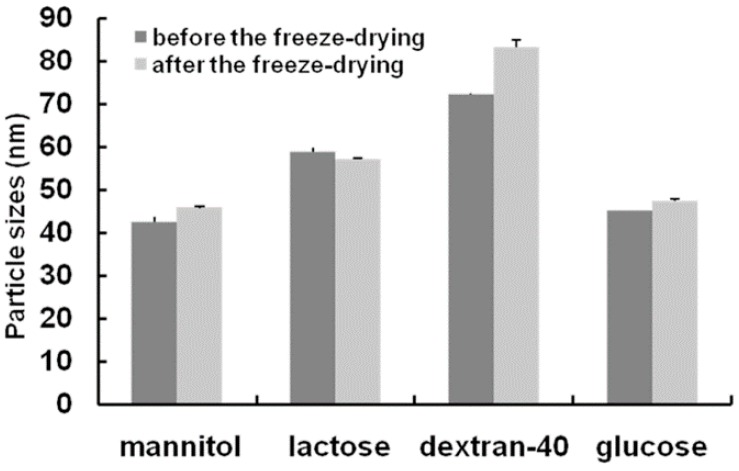
Particle sizes before and after freeze-drying.

Several aqueous vehicles, such as mannitol, lactose, Dextran-40 and glucose, were prepared together with the SMEDDS, in different proportions, as freeze-dried solid carriers. As reported previously, we found that lactose and glucose, which have a relatively small molecular weight and hygroscopicity, were not good carriers for the S-SMEDDS [26]. Comparisons of the particle sizes before and after the freeze-drying process are shown in Figure 3: the particle sizes were within 100 nm after redispersion. As shown in Table 3, drug loading with Dextran-40 as the carrier was higher than with the other carriers. On the basis of the morphology, appearance, hygroscopicity, redispersibility properties and drug loading characteristics, Dextran-40 and mannitol (1:2) were selected as the final aqueous vehicles.

S-SMEDDS were prepared using the optimized formulation, and the contents of six components in the S-SMEDDS were as follows: danshensu 6.01 mg·g^−1^, salvianolic acid B 48.08 mg·g^−1^, dihydrotanshinone I 0.53 mg·g^−1^, tanshinone I 0.47 mg·g^−1^, cryptotanshinone 1.60 mg·g^−1^, tanshinone IIA 2.57 mg·g^−1^.

### 2.2. Dispersibility Test

**Figure 4:**
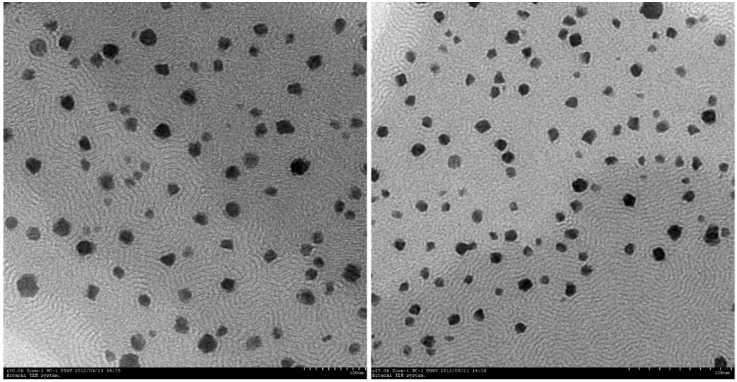
Representative transmission electron microscopy image of the microemulsion. Almost all of the microemulsion was spherical in shape.

A visual test was carried out to assess the self-emulsification of the S-SMEDDS in distilled water at 37 °C, under gentle agitation. The S-SMEDDS showed spontaneous microemulsification, and there were no signs of phase separation or phase inversion of the microemulsion after storage for 2 h. The TEM micrographs (Figure 4) revealed that almost all the microemulsions were spherical in shape. The average droplet size was 59.6 nm and the average zeta potential was −16.9 mv.

### 2.3. Characterization of Solid SMEDDS

**Figure 5:**
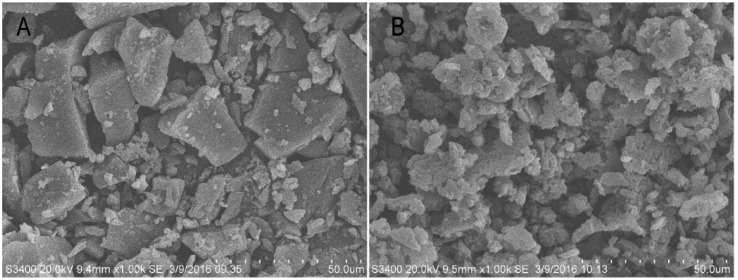
Scanning electron micrographs: (**A**) salvia extract; (**B**) solid SMEDDS.

The scanning electron micrographs of salvia extract and solid SMEDDS formulation are shown in Figure 5. Salvia extract (Figure 5A) appeared as irregular shape particles. The S-SMEDDS (Figure 5B) gave particles with a crushed and loose structure. Our results suggested that the liquid SMEDDS formed a kind of micro-sized microparticles with hydrophilic carrier.

**Figure 6:**
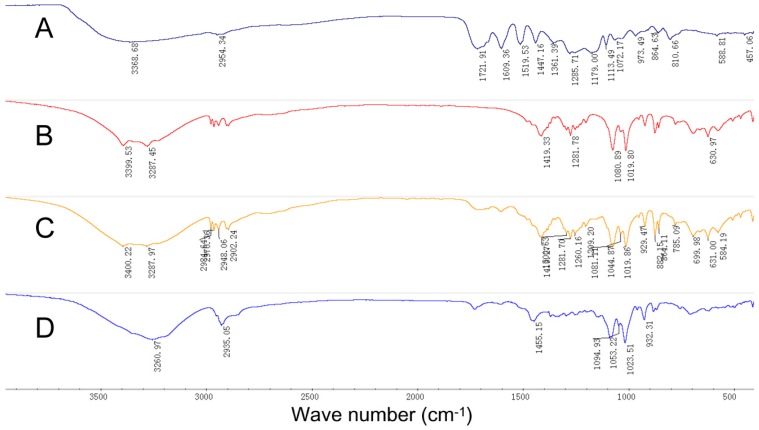
FTIR: (**A**) salvia extract; (**B**) the carriers; (**C**) physical mixture of salvia extract and carriers; (**D**) solid SMEDDS.

The Fourier transform infrared (FTIR) spectra of salvia extract, the carriers, physical mixture of salvia extract and carriers and solid SMEDDS formulations are shown in Figure 6: the spectrogram of solid SMEDDS is similar with the physical mixture, and no new absorption peak appeared (Figure 6D). The results showed that the SMEDDS and the solid carrier can be compatible.

### 2.4. In Vitro Release Study

**Figure 7:**
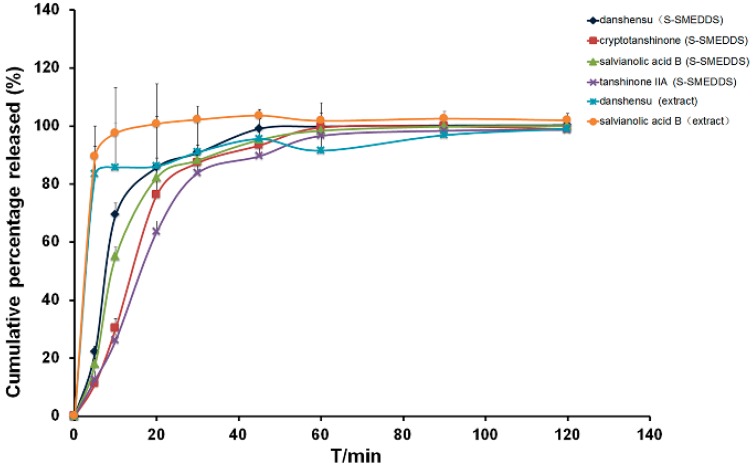
Profiles of the cumulative releases of cryptotanshinone, tanshinone IIA, danshensu and salvianolic acid B from the S-SMEDDS containing a multicomponent mixture of *Salvia miltiorrhiza* extract and unformulated extract (Extract). Between 60% and 80% of each drug analyzed was released from the S-SMEDDS within 20 min.

The *in vitro* release assay (Figure 7) showed that approximately 60% of each of the four drugs assayed (tanshinone IIA, cryptotanshinone, salvianolic acid B and danshensu) was released from the S-SMEDDS within 20 min. The tanshinone IIA and cryptotanshinone released from the extract could not be detected; additionally, danshensu and salvianolic acid B exhibited burst release and more than 80% of them were released from the extract in 5 min (83.40% ± 16.65% of danshensu, 89.56% ± 3.41% of salvianolic acid B) compared to the S-SMEDDS (22.16% ± 2.04% of danshensu, 17.94% ± 1.87% of salvianolic acid B). Thus, S-SMEDDS, one of lipid-based drug delivery systems, can improve the release of multiple components in salvia.

### 2.5. Bioavailability Study

**Figure 8:**
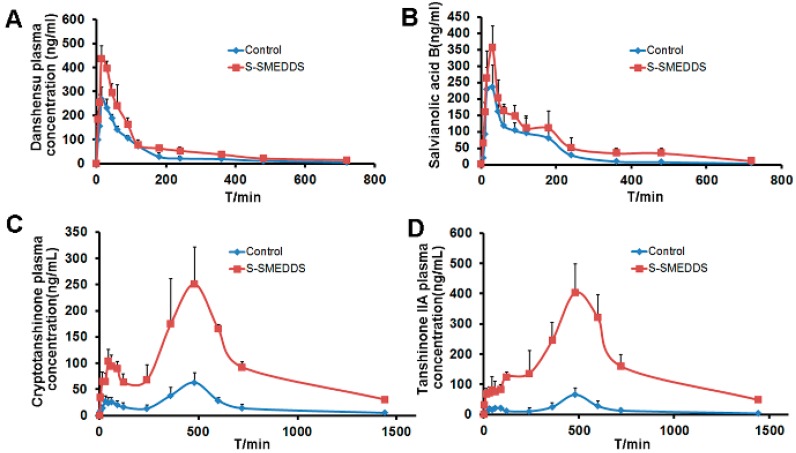
Mean plasma concentration-time curves for danshensu (**A**); salvianolic acid B (**B**); cryptotanshinone (**C**) and tanshinone IIA (**D**), determined after the oral administration of the drugs to rats as either a S-SMEDDS or a suspension (*n* = 6 for each). For all drugs, but particularly for cryptotanshinone and tanshinone IIA, a higher plasma concentration was achieved for the S-SMEDDS than for the suspension.

The mean plasma concentration profiles for tanshinone IIA, cryptotanshinone, salvianolic acid B and danshensu, after oral administration to rats as either a suspension or as a S-SMEDDS, are shown in Figure 8. For all four drugs assayed, the S-SMEDDS containing salvia extract provided a higher plasma concentration of drug than the suspension.

The AUC_0–t_, C_max_, T_max_, T_1/2_ and relative bioavailability for each of the four drugs, administered as either a suspension or as a S-SMEDDS, are listed in Table 4. The relative bioavailability was calculated by dividing the AUC_0–t_ value for the S-SMEDDS preparation by that for the suspension. For each drug, the AUC for the S-SMEDDS was significantly larger than that for the suspension.

The AUC and C_max_ values of tanshinone IIA, cryptotanshinone, salvianolic acid B and danshensu were enhanced after giving developed S-SMEDDS. The increase of C_max_ can be explained by the greater drug absorption of S-SMEDDS. One possibility for enhanced oral absorption is that SMEDDS possesses nano-size particles and provides a large surface area for more absorption. Moreover, the formulation including oil, surfactant and consurfactant can improve solubilization of the active ingredients. Intestinal lipid bilayer membranes may also be affected by the surfactants, resulting in the enhancement of drug permeability [16]. In addition, the oil in SMEDDS increases the amount of lipophilic drug transport through the intestinal lymphatic system which plays an important absorption pathway of poorly water-soluble drugs [27]. Though, of course, further study is needed.

Taken together, these results suggest that the S-SMEDDS may be useful for enhancing the oral absorption of salvia extract. A higher release rate may play an important role in enhancing bioavailability, but it was not the only determinant, especially for the lipophilic ingredients of salvia. Furthermore, encapsulating two kinds of drugs, with differing polarities, into one drug delivery system may enhance the synergistic effects among the various constituents of salvia, and hence the efficacy of the administered drugs.

## 3. Materials and Methods

### 3.1. Ethics Statement

All animal experiments were approved by the Animal Ethics Committee of Nanjing University of Chinese Medicine, Nanjing, China.

### 3.2. Reagents and Chemicals

The reference standards of tanshinone IIA, salvianolic acid B, tanshinone I and cryptotanshinone and internal standard (I.S.) of osthole and ferulic acid were obtained from the National Institutes for Food and Drug Control (Beijing, China). Sodium danshensu and dihydrotanshinone I were sourced from Zelang Medical Technology Co. Ltd. (Nanjing, China). Isopropyl myristate (IPM), Tween-80, PEG400, mannitol, lactose, dextran-40 and glucose were purchased from Sinopharm Chemical Reagent Co. Ltd. (Shanghai, China). Caprylic/capric triglyceride (Labrafac), glyceryl monolinoleate (Maisine 35-1), glyceryl monooleate (Peceol), lauroyl macorgolglycerides (Gelucire44/14), capryloc aproyl macrogol-8 glycerides (Labrasol), oleoyl macrogolglycerides(Labrafil M 1944) and diethylene glycol monoethyl ether (Transcutol P) were obtained from Gattefossé Co. (Lyon, France). Polyoxyl 40 hydrogenated castor oil (Cremophor RH40) and macrogol-15-hydroxystearate (Solutol HS15) were purchased from BASF Co. (Ludwigshafen, Germany). Salvia was purchased from the Jiangsu Province Hospital of TCM and authenticated by Professor Wu (Nanjing University of Chinese Medicine, Nanjing, China). All other chemicals were of analytical grade.

### 3.3. Preparation of Salvia Extract

A liposoluble extract was obtained as described previously [28]. Briefly, the crude drug was extracted by supercritical fluid extraction with two volumes of 95% ethanol as a co-solvent. The extraction conditions were: pressure, 30 MPa; extraction temperature, 45 °C; and time, 2 h. The contents of tanshinone IIA, cryptotanshinone, tanshinone I and dihydrotanshinone I in the liposoluble extract were measured to be 309 mg·g^−1^, 191 mg·g^−1^, 57 mg·g^−1^ and 64 mg·g^−1^, respectively.

An aqueous extract was obtained as follows. The residue of the supercritical fluid extraction was extracted in an acid solution (pH 3) for 1 h, and the filtered solution purified using D101 macroporous resin. After elution with 20% ethanol to remove impurities, the solvent from an elution with 50% ethanol was collected and dried by a vacuum drying method. The content of danshensu was determined to be 72 mg·g^−1^, and that of salvianolic acid B was 576 mg·g^−1^.

### 3.4. Solubility Studies

The solubility of the salvia extract in various oils, surfactants and cosurfactants was determined as follows [29]. Vehicle (1 g) was added to capped tubes containing an equal amount of salvia extract (200 mg). After the test tubes had been sealed, the mixtures in the test tubes were vortex-mixed for min, shaken for 24 h in a water bath maintained at 37 °C, and then centrifuged at 4000 rpm for 10 min. The supernatant was transferred to a 10 mL of volumetric flask, and methanol was added to make up the volume. After filtration through a 0.45-µm membrane filter, the concentrations of 6 constituents (tanshinone IIA, cryptotanshinone, tanshinone I, dihydrotanshinone, danshensu and salvianolic acid B) were determined by ultra-performance liquid chromatography (UPLC). UPLC analysis was carried out using a Waters Acquity UPLC-TM system (Waters Corporation, Milford, MA, USA) and an Acquity UPLC BEH C18 column (50 mm × 2.1 mm, 1.7 µm) at 40 °C. Acetonitrile was used as mobile phase A, and 0.3% formic acid in water was used as mobile phase B. The gradient program was set as follows: from 0 to 1 min, mobile phase A was held at 8%; from 1 to 1.5 min, a linear gradient for mobile phase A from 8% to 20%; from 1.5 to 4 min, mobile phase A was held at 20%; from 4 to 5 min, a linear gradient for mobile phase A from 20% to 60%; from 5 to 9 min, a linear gradient for mobile phase A from 60% to 70%; from 9 to 10 min, a linear gradient for mobile phase A from 70% to 8%; from 10 to 12 min, mobile phase A was held at 8%. The flow rate of the mobile phase was maintained at 0.3 mL/min; the temperature was set at 40 °C; the injection volume was 2 µL; and the detection wavelength was 280 nm.

### 3.5. Phase Diagram Construction

The existence of self-emulsifying oil formulation fields, which could self-emulsify under dilution and gentle agitation, was identified from ternary phase diagrams of systems containing an oil-surfactant-cosurfactant mixture. The ratios of surfactant to cosurfactant ranged from 10:0 to 1:9. These mixtures (S/CoS) were mixed with the oil phase to give weight ratios of 90:10, 80:20, 70:30, 60:40, 50:50, 40:60, 30:70, 20:80 or 10:90. The formulation (1 g) was added to water drop-by-drop and stirred using a magnetic stirrer. The tendency to spontaneously emulsify and the progression of the emulsion droplets were observed. The tendency to form an emulsion was judged as “good” when the droplets easily spread out in water and formed a fine milky emulsion, and “bad” when there was poor or no emulsion formation with the immediate coalescence of oil droplets, especially when stirring was stopped. Phase diagrams were constructed to identify the “good” self-emulsifying region. IPM and Maisine 35-1 (1:1) were used as oil phases, Cremophor RH40 and Solutol HS15 (1:1) as surfactants, and Transcutol P as a cosurfactant. The existence of SMEDDS fields was identified preliminarily by visual observation, as described in *Section 3.7 Preparation of a S-SMEDDS containing a multicomponent mixture of salvia extract* below After dilution and gentle agitation of the mixtures in water, the emulsification behavior was observed. The boundaries of the self-microemulsification regions in the phase diagrams were determined by connecting the points representing formation of the microemulsion.

### 3.6. Screening for Solid Carriers

In this study, mannitol, lactose, Dextran-40 and glucose were assayed as solid carriers. The ratio of liquid SMEDDS to carrier was selected as 1:3, 2:3 or 3:3; all were prepared by freeze-drying. The morphology, appearance and hygroscopicity were observed to judge the adsorptive capacity of these various water-soluble carriers. For the redispersion study, the freeze-drying product (1 g), containing the liquid SMEDDS and carrier at a ratio of 1:3, was diluted with purified water maintained at 37 °C, and stirred at 50 rpm with a rotating magnetic stirrer. The droplet size of the resultant emulsion was determined using a Zetasizer 3000HSA (Malvern Instruments Co. Ltd., Malvern, UK). Each sample was analyzed in triplicate. After the redispersion microemulsion was placed 1 h, the supernatant was transferred to a 10 mL of volumetric flask, and methanol was added to make up the volume. After filtration through a 0.45-µm membrane filter, the concentrations of the 6 constituents of interest were determined by ultra-performance liquid chromatography (UPLC) to investigate the drug loading capabilities of the carriers.

### 3.7. Preparation of a S-SMEDDS Containing a Multicomponent Mixture of Salvia Extract

An aqueous extract was prepared by dissolution in distilled water, together with a mixture of mannitol and dextran-40. The liposoluble extract was prepared by dissolution (with continuous stirring) in a mixture of (IPM+ Maisine 35-1), (Cremophor RH40+ Solutol HS15) and Transcutol P. This mixture was then added to the aqueous phase mixture, and stirred continuously for 10 min at 37 °C so that the dispersion became transparent. Next, the drug-containing microemulsion was placed into penicillin vials, cooled in a refrigerator to 4 °C (for 12 h), and finally prepared by freeze-drying.

### 3.8. Dispersibility Test

The effects of dilution on the S-SMEDDS were investigated, since dilution would be expected to mimic the conditions of the stomach after oral administration. For the dilution study, the S-SMEDDS (500 mg) was introduced into 50 mL of distilled water in a glass beaker, maintained at 37 °C, and the contents mixed gently using a magnetic stirrer. The tendency for spontaneous emulsification to occur and the progression of the emulsion droplets were observed over time. The emulsification of the S-SMEDDS was judged qualitatively to be “good” when a clear microemulsion was formed, and “poor” when a turbid or milky-white emulsion formed after the cessation of stirring [30].

### 3.9. Determination of Droplet Size and Zeta Potential

The formulation (0.5 g) was diluted with purified water maintained at 37 °C, with stirring at 50 rpm using a rotating magnetic stirring apparatus (Gongyi Yuhua Instruments Co. Ltd., Gongyi, Henan, China). The droplet size and the distribution of the resultant emulsion were determined using a Zetasizer 3000HSA (Malvern Instruments Co. Ltd, Malvern, UK). Each sample was analyzed in triplicate. The zeta potential was determined in the same manner.

### 3.10. Determination of Drug Content

The concentrations of six components of S-SMEDDS (tanshinone IIA, cryptotanshinone, tanshinone I, dihydrotanshinone, danshensu and salvianolic acid B) were determined by UPLC. The S-SMEDDS preparation was dissolved in a sufficient quantity of methanol, and the resulting solution was sonicated for 10 min to extract the components, and then filtered. The UPLC technique used was as described above.

### 3.11. Characterization of the Solid SMEDDS

The outer macroscopic structures of salvia extract and solid SMEDDS formulation were examined using a scanning electron microscope (S-3400N, Hitachi, Tokyo, Japan). The powders were fixed to an aluminium stub using double-sided adhesive tape made electrically conductive. The samples were sputter- coated with platinum prior to morphological assessment (E-1010, Hitachi). Furthermore, the spectral characteristics of salvia extract, the carriers, physical mixtures and solid SMEDDS formulations were investigated using Fourier transform infrared spectroscope (Nicolet is5, Thermo, Waltham, MA, USA). The samples were mixed with KBr in dry conditions, pressed into tablets and then analyzed with wave number from 4000 cm^−1^ to 400 cm^−1^.

### 3.12. In Vitro Release Assay

*In vitro* release assays were carried out using methods described in the Chinese Pharmacopeia (2010 edition; “small cup” method) [31]. The S-SMEDDS preparation was added to a capsule (size zero), which was then placed into 250 mL of simulated gastric fluid (0.1 mol·L^−1^ HCL). Samples (2 mL) were removed for analysis at various time points (5, 10, 20, 30, 45, 60, 90 and 120 min), with the volume replaced each time by the addition of 2 mL of fresh medium.

The concentrations of tanshinone IIA, salvianolic acid B, cryptotanshinone and sodium danshensu were measured as described above. The percentage cumulative release was calculated using the following equation:

(1) $$Q=\frac{Ct\times V+\sum_{i=1}^{n-1}C_{t-1}\times Va}{W}\times 100\%$$

where Q is the cumulative percentage released; C_t_ is the concentration of the component at time t; V is the volume of the release medium (V = 250 mL); V_a_ is the volume of the sampling aliquot (V_a_ = 2 mL); and W is the initial amount of component in the capsule.

### 3.13. Bioavailability Study

Male Sprague-Dawley (SD) rats (weight, 200 ± 20 g) were supplied by the Experimental Animal Center of Nanjing University of Chinese Medicine. The rats were divided into two groups of six animals. Prior to drug administration, the animals were fasted for 12 h, with free access to water. The preparations tested were the S-SMEDDS and a drug suspension: the S-SMEDDS was prepared using the method described above, while the suspension was prepared by dispersing salvia extract in sodium carboxymethyl cellulose solution. Rats were given the S-SMEDDS preparation or drug suspension by gavage, such that the following doses were administered: tanshinone IIA, 15.42 mg·kg^−1^; sodium danshensu, 36 mg·kg^−1^; salvianolic acid B, 288 mg·kg^−1^; and cryptotanshinone, 9.6 mg·kg^−1^. Blood samples (0.5 mL) obtained by retro-orbital puncture were collected into heparinized tubes at various time points after administration of the drug preparations: 5, 10, 15, 30, 45, 60, 90, 120, 240, 360, 480, 600, 720 and 1440 min. The blood samples were centrifuged at 5000 r·min^−1^ for 5 min to obtain plasma, which was stored at −20 °C until required for analysis (described below).

For analysis of water-soluble drugs, a 100-µL sample of plasma was vortex-mixed with 20 µL of 2% perchloric acid solution; this was then vortex-mixed with 1 mL of ethyl acetate. After centrifugation at 10,000 rpm for 5 min, 1 mL of the upper layer was removed and dried under gently flowing nitrogen. The residue was reconstituted in 100 µL of 70% methanol, vortex-mixed for 2 min, and then centrifuged at 10,000 rpm for 5 min. The supernatant was used for analysis of content.

For analysis of fat-soluble drugs, a 100-µL sample of plasma was vortex-mixed for 2 min with 0.4 mL of acetonitrile. After centrifugation at 10,000 rpm for 5 min, 0.4 mL of the upper layer was removed and dried under gently flowing nitrogen. The residue was reconstituted in 100 µL of 70% methanol, vortex-mixed for 2 min, and then centrifuged at 10,000 rpm for 5 min. The supernatant was used for analysis of content.

The concentrations of tanshinone IIA and cryptotanshinone in rat plasma were determined using a Waters XevoTQD UPLC-MS system (Waters Corporation) and an Acquity UPLC R BEH C18 column (1.7 µm, 2.1 × 50 mm) at 40 °C. The mobile phase was a mixture of acetonitrile and 0.3% formic acid solution (70:30); and the flow rate was 0.3 mL·min^−1^. Triple-quadruple tandem mass spectrometric detection was carried out in positive ionization mode, with a source temperature of 150 °C, a scan mode of MRM, a desolvation flow rate of 800 L/h, and a desolvation temperature of 500 °C.

The concentrations of salvianolic acid B and sodium danshensu in rat plasma were determined using the UPLC-MS system and column described above. Mobile phase A was acetonitrile, and mobile phase B was 0.1% formic acid in water, with a gradient program set as follows: 0 to 0.5 min, a linear gradient for mobile phase A from 5% to 25%; 0.5 to 2 min, mobile phase A was held at 25%; 2 to 2.5 min, a linear gradient for mobile phase A from 25% to 5%; 2.5 to 5 min, mobile phase A was held at 5%. The flow rate was 0.4 mL·min^−1^. Triple-quadruple tandem mass spectrometric detection was operated in positive ionization mode, with a source temperature of 150 °C, a scan mode of SIR, a desolvation flow rate of 1000 L/h, and a desolvation temperature of 550 °C.

### 3.14. Data Analysis

Pharmacokinetics parameters were obtained using Drug and Statistics for Windows (DAS) 3.2.2 software (Chinese Pharmacological Society, Beijing, China). The following parameters were determined: the mean area under the curve of the drug concentration-time profile (AUC_0–t_), peak plasma concentration (C_max_), time of peak plasma concentration (T_max_) and half-life (T_1/2_). The relative bioavailability was calculated by dividing the AUC_0–t_ value for the S-SMEDDS by that for the suspension, and converting the ratio to a percentage. Statistical analysis of the pharmacokinetics parameters was carried out using a non-compartment model. A value of *p* ≤ 0.05 was considered statistically significant.

## 4. Conclusions

In conclusion, the novel S-SMEDDS developed in this study increased the dissolution rate and improved the oral absorption of two types of drug with differing polarities. We propose that S-SMEDDS is a promising new method by which to deliver salvia extract and potentially other multicomponent drugs by the oral route.

## Figures and Tables

**Table 1:** Solubilities of 6 components of salvia in various vehicles at 37 °C.

Vehicle		Danshensu (mg·g^−1^)	Salvianolic Acid B (mg·g^−1^)	Dihydrotanshinone I (mg·g^−1^)	Cryptotanshinone (mg·g^−1^)	Tanshinone I (mg·g^−1^)	Tanshinone IIA (mg·g^−1^)
Oils	IPM	-	-	0.388 ± 0.035	0.226 ± 0.013	1.757 ± 0.062	4.525 ± 0.026
	Maisine 35-1	3.493 ± 0.015	2.350 ± 0.136	0.513 ± 0.014	0.101 ± 0.021	1.365 ± 0.042	4.123 ± 0.018
	Labrafac	-	-	-	0.301 ± 0.038	1.191 ± 0.017	3.783 ± 0.121
	Peceol	-	-	0.377 ± 0.012	-	1.179 ± 0.023	2.742 ± 0.029
Surfactants	Cremophor RH40	1.188 ± 0.023	3.439 ± 0.103	-	0.399 ± 0.047	1.497 ± 0.037	4.094 ± 0.079
	Labrafil M 1944	-	-	0.442 ± 0.110	-	1.283 ± 0.017	3.805 ± 0.169
	Solutol HS15	0.677 ± 0.126	-	0.136 ± 0.009	0.197 ± 0.027	1.527 ± 0.028	3.760 ± 0.321
	Labrasol	1.629 ± 0.220	1.783 ± 0.209	0.307 ± 0.052	0.127 ± 0.017	1.405 ± 0.175	3.323 ± 0.261
	Tween-80	1.624 ± 0.158	1.155 ± 0.067	-	0.204 ± 0.046	-	3.241 ± 0.333
	Gelucire 44/14	0.813 ± 0.216	0.733 ± 0.08	0.428 ± 0.099	-	1.139 ± 0.114	2.035 ± 0.07
Cosurfactants	Transcutol P	2.410 ± 0.202	6.480 ± 0.258	0.888 ± 0.036	0.663 ± 0.114	2.367 ± 0.136	3.579 ± 0.133
	Ethanol	0.961 ± 0.151	3.325 ± 0.143	0.499 ± 0.02	-	1.229 ± 0.108	2.022 ± 0.087
	PEG400	0.309 ± 0.037	4.004 ± 0.027	-	0.260 ± 0.041	1.277 ± 0.117	2.429 ± 0.125

Abbreviations: IPM, Isopropyl myristate; PEG 400, Polyethylene glycol 400.

**Table 2:** Droplet size, polydispersibility and solubility of the 5 SMEDDS formulations tested.

Formulation Composition (35:45:20 *w*/*w*/*w*)	Droplet Size (nm)	Polydispersibility	Solubility					
			Danshensu (mg·g^−1^)	Salvianolic Acid B (mg·g^−1^)	Dihydrotanshinone I (mg·g^−1^)	Tanshinone I (mg·g^−1^)	Cryptotanshinone (mg·g^−1^)	Tanshinone IIA (mg·g^−1^)
A	64.60	0.136	0.34 ± 0.07	0.49 ± 0.07	1.50 ± 0.29	1.46 ± 0.12	4.64 ± 0.29	5.14 ± 0.12
B	67.20	0.176	0.57 ± 0.12	0.62 ± 0.10	1.43 ± 0.13	1.39 ± 0.15	4.28 ± 0.28	5.30 ± 0.18
C	102.30	0.264	1.75 ± 0.32	2.03 ± 0.05	1.25 ± 0.09	1.45 ± 0.30	4.05 ± 0.39	4.59 ± 0.29
D	91.56	0.390	1.51 ± 0.09	2.31 ± 0.17	1.33 ± 0.20	1.37 ± 0.16	4.60 ± 0.40	4.67 ± 0.23
E	49.20	0.073	1.37 ± 0.11	1.11 ± 0.20	1.42 ± 0.36	1.40 ± 0.24	4.65 ± 0.32	5.16 ± 0.17

A: IPM, Cremophor RH40 and Transcutol P; B: IPM, Solutol HS15 and Transcutol P; C: Maisine 35-1, Solutol HS15 and Transcutol P; D: Maisine 35-1, Cremophor RH40 and Transcutol P; E: (IPM+ Maisine 35-1), (Cremophor RH40+ Solutol HS15) and Transcutol P.

**Table 3:** Loading of the 6 components of salvia in various water-soluble carriers (*n* = 3).

Carrier	Danshensu (mg·g^−1^)	Salvianolic Acid B (mg·g^−1^)	Dihydrotanshinone I (mg·g^−1^)	Tanshinone I (mg·g^−1^)	Cryptotanshinone (mg·g^−1^)	Tanshinone IIA (mg·g^−1^)
Mannitol	1.61 ± 0.06	13.88 ± 0.30	0.20 ± 0.09	0.21 ± 0.06	0.64 ± 0.14	1.03 ± 0.14
Lactose	1.47 ± 0.12	13.53 ± 0.19	0.20 ± 0.04	0.20 ± 0.01	0.60 ± 0.10	0.94 ± 0.05
Dextran-40	1.63 ± 0.07	14.30 ± 0.19	0.21 ± 0.01	0.23 ± 0.03	0.71 ± 0.12	1.14 ± 0.12
Glucose	1.49 ± 0.11	13.45 ± 0.29	0.18 ± 0.02	0.20 ± 0.02	0.59 ± 0.04	0.89 ± 0.01

**Table 4:** Comparisons of pharmacokinetics parameters between the S-SMEDDS and a drug suspension.

Formulation	C_max_ (ng·mL^−1^)	T_max_ (h)	T_1/2_ (h)	AUC_0–t_ (μg·h·mL^−1^)	Relative Bioavailability (%)
Danshensu, suspension	278.84 ± 32.52	0.29 ± 0.10	3.39 ± 0.95	471.51 ± 52.11	
Danshensu, S-SMEDDS	451.82 ± 30.22 ***	0.33 ± 0.13	3.56 ± 1.24	822.43 ± 91.70 ***	174.4
Salvianolic acid B, suspension	285.09 ± 59.04	0.38 ± 0.14	1.95 ± 0.87	484.02 ± 65.14	
Salvianolic acid B, S-SMEDDS	369.41 ± 65.85 *	0.46 ± 0.10	3.63 ± 1.001 *	801.23 ± 148.55 **	165.5
Cryptotanshinone, suspension	63.38 ± 12.05	8.00 ± 0.00	3.33 ± 2.29	7.92 ± 2.31	
Cryptotanshinone, S-SMEDDS	270.42 ± 63.47 ***	8.40 ± 0.89	5.02 ± 1.78	39.52 ± 9.91 ***	499.3
Tanshinone IIA, suspension	65.83 ± 20.24	8.00 ± 0.00	4.11 ± 2.06	6.93 ± 0.92	
Tanshinone IIA, S-SMEDDS	403.34 ± 90.71 ***	8.00 ± 0.00	4.71 ± 0.87	65.04 ± 14.07 ***	938.2

Mean ± S.D., *n* = 6. * *p* < 0.05, ** *p* < 0.01, *** *p* < 0.001 *versus* oral administration of suspension.Each value is presented as the mean ± standard deviation (*n* = 6). *C*_max_, peak plasma concentration; T_max_, time of peak plasma concentration; T_1/2_, half-life; AUC_0–t_, mean area under the curve of the drug concentration-time profile. The relative bioavailability was calculated by dividing the AUC_0–t_ value for the S-SMEDDS by that for the suspension, and converting the ratio to a percentage.

## References

[1] Yang L., Ding G., Lin H., Cheng H., Kong Y., Wei Y., Fang X., Liu R., Wang L., Chen X. (2013). Transcriptome analysis of medicinal plant Salvia miltiorrhiza and identification of genes related to tanshinone biosynthesis. PLoS ONE.

[2] Chen X., Guo J., Bao J., Lu J., Wang Y. (2014). The anticancer properties of salvia miltiorrhiza Bunge (Danshen): A systematic review. Med. Res. Rev..

[3] Jiang W.Y., Jeon B.H., Kim Y.C., Lee S.H., Sohn D.H., Seo G.S. (2013). PF2401-SF, standardized fraction of Salvia miltiorrhiza shows anti-inflammatory activity in macrophages and acute arthritis *in vivo*. Int. Immunopharmacol..

[4] Shen W., Zhang Y., Li W., Cong J., Zhou Y., Ng E.H., Wu X. (2013). Effects of tanshinone on hyperandrogenism and the quality of life in women with polycystic ovary syndrome: Protocol of a double-blind, placebo-controlled, randomised trial. BMJ Open.

[5] Zhou L., Zuo Z., Chow M.S. (2005). Danshen: An overview of its chemistry, pharmacology, pharmacokinetics, and clinical use. J. Clin. Pharmacol..

[6] Kim H.K., Woo E.R., Lee H.W., Park H.R., Kim H.N., Jung Y.K., Choi J.Y., Chae S.W., Kim H.R., Chae H.J. (2008). The correlation of Salvia miltiorrhiza extract-induced regulation of osteoclastogenesis with the amount of components tanshinone I, tanshinone IIA, cryptotanshinone, and dihydrotanshinone. Immunopharmacol. Immunotoxicol..

[7] Lu Y., Liu X., Liang X., Xiang L., Zhang W. (2011). Metabolomic strategy to study therapeutic and synergistic effects of tanshinone IIA, salvianolic acid B and ginsenoside Rb1 in myocardial ischemia rats. J. Ethnopharmacol..

[8] Chong Y., Wang T., Wang W., Zhang L., Li C., Yu P., Wang H., Fu F. (2012). Down-regulation of P-glycoprotein expression contributes to an increase in Danshensu accumulation in the cerebral ischemia/reperfusion brain. Mol. Med. Rep..

[9] Hao H., Wang G., Cui N., Li J., Xie L., Ding Z. (2006). Pharmacokinetics, absorption and tissue distribution of tanshinone IIA solid dispersion. Planta Med..

[10] Zhang J., Huang M., Guan S., Bi H.C., Pan Y., Duan W., Chan S.Y., Chen X., Hong Y.H., Bian J.S. (2006). A mechanistic study of the intestinal absorption of cryptotanshinone, the major active constituent of Salvia miltiorrhiza. J. Pharmacol. Exp. Ther..

[11] Zhou L., Chow M.S., Zuo Z. (2009). Effect of sodium caprate on the oral absorptions of danshensu and salvianolic acid B. Int. J. Pharm..

[12] Pan Y., Bi H.C., Zhong G.P., Chen X., Zuo Z., Zhao L.Z., Gu L.Q., Liu P.Q., Huang Z.Y., Zhou S.F. (2008). Pharmacokinetic characterization of hydroxylpropyl-β-cyclodextrin-included complex of cryptotanshinone, an investigational cardiovascular drug purified from Danshen (*Salvia miltiorrhiza*). Xenobiotica.

[13] Li J., Liu P., Liu J.P., Zhang W.L., Yang J.K., Fan Y.Q. (2012). Novel Tanshinone II A ternary solid dispersion pellets prepared by a single-step technique: *In vitro* and *in vivo* evaluation. Eur. J. Pharm. Biopharm..

[14] Li J., Liu P., Liu J.P., Yang J.K., Zhang W.L., Fan Y.Q., Kan S.L., Cui Y., Zhang W.J. (2013). Bioavailability and foam cells permeability enhancement of Salvianolic acid B pellets based on drug-phospholipids complex technique. Eur. J. Pharm. Biopharm..

[15] Peng Q., Gong T., Zuo J., Liu J., Zhao D., Zhang Z. (2008). Enhanced oral bioavailability of salvianolic acid B by phospholipid complex loaded nanoparticles. Die Pharm..

[16] Gursoy R.N., Benita S. (2004). Self-emulsifying drug delivery systems (SEDDS) for improved oral delivery of lipophilic drugs. Biomed. Pharmacother..

[17] O’Driscoll C.M. (2002). Lipid-based formulations for intestinal lymphatic delivery. Eur. J. Pharm. Sci..

[18] Hauss D.J., Mehta S.C., Radebaugh G.W. (1994). Targeted lymphatic transport and modified systemic distribution of CI-976, a lipophilic lipid-regulator drug, via a formulation approach. Int. J. Pharm..

[19] Patel A.R., Vavia P.R. (2007). Preparation and *in vivo* evaluation of SMEDDS (self-microemulsifying drug delivery system) containing fenofibrate. AAPS J..

[20] Tuleu C., Newton M., Rose J., Euler D., Saklatvala R., Clarke A., Booth S. (2004). Comparative bioavailability study in dogs of a self-emulsifying formulation of progesterone presented in a pellet and liquid form compared with an aqueous suspension of progesterone. J. Pharm. Sci..

[21] Nazzal S., Khan M. (2006). Controlled release of a self-emulsifying formulation from a tablet dosage form: Stability assessment and optimization of some processing parameters. Int. J. Pharm..

[22] Wang L., Dong J., Chen J., Eastoe J., Li X. (2009). Design and optimization of a new self-nanoemulsifying drug delivery system. J. Colloid Interface Sci..

[23] Tang B., Cheng G., Gu J.C., Xu C.H. (2008). Development of solid self-emulsifying drug delivery systems: Preparation techniques and dosage forms. Drug Discov. Today.

[24] Rao S.V.R., Shao J. (2008). Self-nanoemulsifying drug delivery systems (SMEDDS) for oral delivery of protein drugs: I. Formulation development. Int. J. Pharm..

[25] Li P., Ghosh A., Wagner R.F., Krill S., Joshi Y.M., Serajuddin A.T. (2005). Effect of combined use of nonionic surfactant on formation of oil-in-water microemulsions. Int. J. Pharm..

[26] Gauthier R.J., Lebinson R.S. (1990). Lyophilized Emulsion Composition and Method.

[27] Dixit A.R., Rajput S.J., Patel S.G. (2010). Preparation and bioavailability assessment of SMEDDS containing valsartan. AAPS PharmSciTech.

[28] Li Y.C., Zeng J.Q., Liu L.M., Jin X.S. (2002). Extraction of three tanshinones from the root of *Salvia miltiorrhiza* Bunge by supercritical carbon dioxide fluid and their analysis with high performance liquid chromatography. Chin. J. Chromatogr..

[29] Chen Z.Q., Liu Y., Zhao J.H., Wang L., Feng N.P. (2012). Improved oral bioavailability of poorly water-soluble indirubin by a supersaturatable self-microemulsifying drug delivery system. Int. J. Nanomed..

[30] Nekkanti V., Karatgi P., Prabhu R., Pillai R. (2010). Solid self-microemulsifying formulation for candesartan cilexetil. AAPS PharmSciTech.

[31] Yang F., Bi G.W., Long X.Y., Yu Z.M., Yang W.S., Feng L.P. (2005). The preparation and the *in vitro* release of OANO-1 microspheres. Chin. J. Chin. Mater. Med.

